# Prevalence and characteristics of persistent taste and smell dysfunction after immune checkpoint inhibitor therapy for cancer

**DOI:** 10.1007/s00520-026-10761-4

**Published:** 2026-05-21

**Authors:** Jip M. van Elst, Corine M Buffinga, Henk S. Brand, Lucie B. M. Hijmering-Kappelle, Harriët Jager-Wittenaar, Anna K. L. Reyners, Janine Nuver, Jacco J. de Haan

**Affiliations:** 1 Department of Medical Oncology, University Medical Center Groningen, University of Groningen, Hanzeplein 1, 9713 GZ Groningen, the Netherlands; 2 Department of Oral Biochemistry, Academic Centre for Dentistry Amsterdam (ACTA), VU University of Amsterdam, University of Amsterdam, Gustav Mahlerlaan 3004, 1081 LA Amsterdam, the Netherlands; 3 Department of Biochemistry, Dental Faculty, Carol Davila University of Medicine and Pharmacy, Bucharest, Romania; 4 Department of Pulmonary Medicine, University Medical Center Groningen, University of Groningen, Hanzeplein 1, 9713 GZ Groningen, the Netherlands; 5 Research Group Healthy Ageing, Allied Health Care and Nursing, Hanze University of Applied Sciences, Petrus Driessenstraat 3, 9714 CA Groningen, the Netherlands; 6 Department of Gastroenterology and Hepatology, Dietetics, Radboud University Medical Center, Geert Grooteplein Zuid 10, 6525 GA Nijmegen, the Netherlands; 7 Faculty of Physical Education and Physiotherapy, Department of Physiotherapy, Human Physiology and Anatomy, Research Unit Experimental Anatomy, Vrije Universiteit Brussel, Laarbeeklaan 103, 1090 Brussels, Belgium

**Keywords:** Immune checkpoint inhibitors, Taste, Smell, Saliva, Oncology, Oral health

## Abstract

**Purpose:**

To determine the prevalence and characteristics of persistent dysfunction of taste and smell, and salivary parameters in patients after completion of ICI therapy.

**Methods:**

In this cross-sectional study, dysfunction in patients treated for cancer with ICIs was compared with dysfunction in caregivers. Subjective taste and smell dysfunction and their impact on life were evaluated using validated questionnaires. Objective taste and smell were assessed with taste strips and Sniffin’ Sticks, and flow rate and biochemical composition of saliva were measured.

**Results:**

A total of 50 patients and 51 caregivers were included. General characteristics of patients did not differ significantly from those of caregivers. Patients had received a median of 14 ICI cycles. The median time since the last ICI cycle was 3.7 years. Six patients (12%) reported mild subjective taste alterations and two (4%) moderate taste alterations, while two caregivers (4%) reported mild taste alterations. Patients scored lower on the appetite subscale and higher on sodium concentration compared to caregivers (respectively, p = 0.017 and p = 0.027). Objective taste and smell function, (un)stimulated saliva flow rates, and xerostomia did not significantly differ between both groups.

**Conclusions:**

These findings suggest that most patients do not experience persistent taste and smell dysfunction, and salivary changes after ICI treatment. However, a subgroup reports symptoms. As subjective experiences may not correspond with objective findings, clinicians should actively inquire about perceived sensory dysfunction as these can impact QoL.

**Trial registration number:** NCT06495008 / 2024–01-02.

**Supplementary Information:**

The online version contains supplementary material available at 10.1007/s00520-026-10761-4.

## Introduction

Immune checkpoint inhibitors (ICIs) are widely used to treat multiple cancer types. These cancer types include melanoma, non-small cell lung cancer (NSCLC), and urogenital cancer [1]. ICIs enhance anti-tumor immunity by blocking inhibitory signals that prevent T cells from attacking cancer cells [2]. Key targets of ICIs include cytotoxic T-lymphocyte antigen-4 (CTLA-4), Programmed death 1 (PD-1) and its ligand (PD-L1) [3].

Long-term survival in patients receiving ICIs is becoming more common as treatment efficacy continues to improve. For instance, ICIs have improved 5-year survival in patients with metastastic melanoma from < 10% (pre-ICI era) to > 50% [4, 5] ICIs are also increasingly used in the adjuvant and neoadjuvant setting [6–8]. Unfortunately, ICIs are associated with immune-related adverse events (AEs), which can affect various organ systems and range from mild to life-threatening [9]. Most commonly affected are the gastrointestinal tract, skin, endocrine glands, and liver [10]. Given the prolonged survival of ICI-treated patients, chronic AEs are becoming increasingly relevant [11]. Chronic AEs are observed in more than 40% of patients [12]. Patients with chronic AEs report reduced quality of life (QoL) [6].

Oral side effects have also been described [13, 14]. One study indicated that 58% of ICI-treated patients with cancer experience xerostomia, 44% develop oral mucosal disorders, and 28% report dysgeusia [15]. However, studies that did not specifically focus on oral side effects report lower prevalence rates [13]. A meta-analysis reported a pooled prevalence of 5% for xerostomia and 3% for dysgeusia among patients with cancer treated with ICIs [16]. Taste and smell alterations usually occur in the first months of ICI treatment, but can also emerge after years [15, 17]. It is important to note that in many studies it remains unclear whether oral side effects were systematically assessed or spontaneously reported by patients. Oral side effects are often neglected by both patients and healthcare professionals. Possibly this is due to underestimation of their clinical significance and a focus on more immediate or life-threatening concerns. Therefore, oral side effects may be easily underreported if not structurally surveyed among patients [18]. An objective determination of taste and smell dysfunction and their prevalence after treatment with ICIs is still lacking.

T cell-mediated inflammation of the salivary glands has been described as a chronic AE of ICI treatment [19]. Irreversible damage to the salivary gland could lead to diminished saliva production and secretion [6]. Oral dryness can contribute to taste disturbances, as saliva is essential for dissolving food and enabling taste molecules to bind to gustatory receptor cells [20]. Furthermore, hyposalivation is a risk factor for oral diseases, including dental caries, oral infections and oral mucosal discomfort or pain [21]. Saliva contains electrolytes and proteins that are essential for antimicrobial defense, food digestion, and wound healing. Changes in saliva composition may therefore contribute to both oral disease and taste disturbances [20, 22]. However, saliva analysis has not been performed in patients treated with ICIs.

Taste and smell dysfunction, xerostomia, and oral mucosal disorders can have a significant impact on QoL, affecting nutritional intake, social interactions, and even household tasks [23–25]. More insight is needed into the prevalence and impact of long-term taste and smell alterations and oral dryness in patients treated with ICIs. These insights could raise awareness of this problem among healthcare providers and help patients cope with these AEs.

This study aimed to determine both the prevalence and characteristics of persistent dysfunction of taste and smell, and secretion rate and composition of saliva after completion of ICI treatment.

## Methods

### Study design and study population

This observational cross-sectional study was carried out between October 2023 and February 2025. Patients aged 18 years or older who had been treated between 2014 and 2022 with ICIs for melanoma or urogenital cancer at the department of Medical Oncology, or NSCLC at the department of Pulmonary Medicine at the University Medical Center Groningen (UMCG) were included. The Medical Ethical Committee of the UMCG approved this study (METc no. 2022/536). This study complied with the Declaration of Helsinki for Medical Research involving Human Subjects and is registered at ClinicalTrials.gov (NCT06495008).

Patients were eligible if they had completed their last cycle of ICI therapy at least two years before inclusion and were able to comply with study procedures. Non-professional caregivers from the patient’s personal environment, aged 18 years or older, served as a control group. Including caregivers in the control group ensured a similar background and thereby improved comparability between groups. However, not all patients had a caregiver who consented to participate. Therefore, caregivers from patients who did not participate in this study were included. These caregivers were mainly partners and relatives of the patients treated for cancer.

Patients and caregivers were excluded if they met the following criteria: 1) Previous or subsequent anti-cancer therapies, except for surgery and palliative radiotherapy outside the head-neck and brain regions; 2) A history of malignancies within the past ten years, excluding non-melanoma skin cancer, cervical intra-epithelial neoplasia, or carcinoma in situ of the breast (for patients: excluding the malignancy for which they were treated with ICIs); 3) A history of disease(s) in the ear, nose or throat, autoimmune disorders, or the use of medication affecting taste, smell, oral mucosa, or saliva production (for patients: before starting ICI therapy). The presence of other comorbidities did not lead to study exclusion and were did not asked for specifically.

### Procedure

Potential participants received an invitation letter explaining the objectives and procedures of the study. Those who agreed to participate were scheduled for a single visit at the UMCG. Assessments took place in a quiet room. During this visit, potential participants could ask questions, and written informed consent was obtained before data collection. Saliva samples were collected, followed by standardized taste and smell tests and the completion of questionnaires assessing perceived taste and smell dysfunction, xerostomia, and quality of life. Additionally, relevant medical and demographic data, including age, sex, medical history, and medication use, were retrieved from the patients’ electronic medical records. Caregivers provided this data through self-reporting.

Saliva collection was performed under standardized conditions [26]. Participants were instructed to refrain from smoking, eating, drinking (except water), or brushing their teeth for at least one hour before collection. First, unstimulated whole saliva was collected. Participants were asked to spit into a pre-weighed container every 30–60 s for five minutes while avoiding swallowing, talking and moving excessively. After a five minute break, stimulated whole saliva was collected, during which participants were asked to chew a 5 × 5 cm piece of parafilm (Parafilm M, Pecheney, Chicago, IL) at a frequency of one chew per second, spitting into a second container at 30–60 s intervals for another five minutes. The tubes with collected saliva were re-weighed to determine the flow rate (mL/min), assuming a density of 1.0 g/mL. The samples were immediately stored at −80 °C for later biochemical analysis. Hyposalivation was defined as an unstimulated salivary flow rate of < 0.1 ml/min and/or a stimulated salivary flow rate of < 0.5 ml/min [27].

Taste and smell function were objectively assessed using validated taste strips and Sniffin’ Sticks [28, 29]. Taste strips consist of filter paper strips impregnated with solutions representing the four basic tastes (sweet, sour, salty and bitter) in four different concentrations, resulting in a total of 16 tests. Each strip was placed on the participant’s tongue, and they were asked to identify the taste. The total score ranged from 0 to 16, with hypogeusia defined as a score below 9, according to the manufacturer’s guidelines. Smell function was assessed using “Sniffin’ Sticks”, measuring both smell identification and smell threshold. For smell identification, 16 odors were presented, and participants selected the correct answer from four multiple-choice options. A score below 11 was considered indicative of hyposmia [30]. Smell threshold was determined using a stepwise method with 16 triplets of pens, one of each triplet containing n-butanol in varying concentrations. Participants identified the pen containing the odorant, and the threshold score was calculated based on the average of the last four reversal points. A score below 5.8 indicated hyposmia [31].

To assess subjective taste and smell perception, as well as the impact of dysfunction on quality of life, participants completed several validated questionnaires. Taste dysfunction was measured using the Dutch version of the Chemotherapy-induced Taste Alteration Scale (CiTAS), which consists of 18 items scored on a five-point Likert scale, where higher scores indicate more pronounced taste alterations. The questionnaire comprises four subscales: reduction in basic tastes, discomfort, phantogeusia or parageusia, and general taste changes. The score of each subscale is derived from the relevant questions that assess those specific aspects of taste dysfunction. The total score is calculated by averaging the scores for all 18 questions. The scores are interpreted as follows: a score lower than 6 indicates no significant taste alterations, 6–9 indicates mild taste changes, 10–14 indicates moderate taste changes, and 15–20 indicates severe taste alterations [32].

The Dutch version of the Patient-Generated Subjective Global Assessment Short Form (PG-SGA SF) was used to assess weight loss, food intake, nutrition impact symptoms, and activities and functioning, with a numerical scoring ranging from 0 to 36. Risk of malnutrition was classified as low (0–3 points), medium (4–8 points), or high (≥ 9 points) [33, 34].

The Appetite, Hunger, and Sensory Perception (AHSP) questionnaire was used to evaluate these aspects. The AHSP consists of 29 multiple-choice questions, all rated on a 5-point Likert scale. The questionnaire includes four subscales: taste (8 questions, score range 8–40), appetite (6 questions, score range 6–30), smell (6 questions, score range 6–30), and hunger (9 questions, score range 9–45) [35].

Quality of life related to smell (and taste) dysfunction was assessed using the Questionnaire of Olfactory Disorders (QOD), which consists of 32 multiple-choice questions. The analysis of the QOD was limited to those individuals who indicated some degree of taste and/or smell changes in the AHSP questionnaire (questions S1, S2, S3, S8, G15, G16, G17, G19, G20). This approach was chosen because individuals without such changes have difficulty assessing the impact on their quality of life, leading to inconsistent or unreliable responses [36].

Xerostomia was evaluated using the Xerostomia Inventory (XI), an 11-item 5-point Likert scale questionnaire, and the Regional Oral Dryness Inventory (RODI), in which participants rated the severity of oral dryness in different intra-oral areas on a 5-point Likert scale [37, 38].

### Biochemical analysis saliva

Prior to analysis, all unstimulated whole saliva samples were centrifuged at 10,000 g for ten minutes at 4 °C. The concentrations of sodium, potassium, ammonium, magnesium, and calcium (mg/L) were determined using capillary electrophoresis with the CAPEL-205 system (Lumex Instruments) [39]. Total protein concentration (µg/mL) was measured using a bicinchoninic acid (BCA) assay, and mucin 5B levels (mg/mL) were quantified using a modified ELISA protocol employing the MUC5B-specific monoclonal antibody F2 [40]. The α-amylase activity (U/mL) was determined enzymatically using a chromogenic substrate assay based on 2-chloro-4-nitrophenyl-α-D-maltotrioside [41].

### Data analysis

Based on previous studies, the prevalence of long-term taste and smell dysfunction among cancer survivors was estimated at 25% [15]. A prevalence of 5% was expected in caregivers, as those with known taste or smell disorders were excluded, but a small group could unknowingly have taste or smell disorders. Based on a sample size calculation, it was determined that a total of 49 patients and 49 caregivers would be required to detect significant differences, with a 95% confidence level and 80% power. Non-normally distributed continuous variables were reported as medians with ranges, while normally distributed continuous variables were reported as mean with standard deviation. Categorical data were presented as percentages.

Normally distributed continuous variables were compared using independent t-tests, while non-normally distributed continuous variables were compared using Mann–Whitney U tests. Categorical variables were analyzed using Chi-square tests. For salivary components, one-tailed t-tests were used based on the hypothesis of a directional effect. Correlations between subjective and objective parameters of taste and smell dysfunction were analyzed using Spearman’s rho, assuming a non-parametric distribution.

## Results

### Study population

In total, 50 patients and 51 caregivers participated in this study. One patient who was also treated with a targeted anti-cancer agent was excluded. Two caregivers were excluded because of comorbidities that could affect taste and smell (olfactory disease and Parkinson’s disease). Of the included patients, 43% (*n* = 21) were female, and 51% (*n *= 25) were former smokers, the mean age was 67.2 years (SD ± 10.7), 78% (*n* = 38) had melanoma, and 37% (*n* = 18) was treated with nivolumab, with a median time of 3.7 years [IQR 2.9–5.1] since their last ICI cycle. Twelve of the 49 patients (24%) received a (different) second type of ICI. Of the caregivers, 53% (*n *= 26) were female, 51% (*n* = 25) were former smokers, and they had a mean age of 64.2 years (SD ± 12.1). No significant differences were found between the two groups regarding gender, age, BMI, or smoking. Table 1 summarizes the characteristics of the study population.

**Table 1:** Characteristics of the study population

Characteristics	Patients n = 49	Caregivers n = 49	P-value
Gender, n (%)			0.419
*Female*	21 (42.9%)	26 (53.1%)	
Age (years), mean ± SD	67.2 ± 10.7	64.2 ± 12.1	0.200
BMI, median [IQR]	26.2 [22.6–29.4]	24.8 [22.5–29.1]	0.541
Smoking, n (%)			0.181
*No*	17 (34.7%)	22 (44.9%)	
*Yes*	7 (14.3%)	2 (4.1%)	
*Former smoker*	25 (51%)	25 (51%)	
Type of tumor, n (%)			
*Melanoma*	38 (77.6%)		
*NSCLC*	5 (10.2%)		
*RCC*	4 (8.2%)		
*UCC*	2 (4.1%)		
ICI therapy, n (%)			
*Nivolumab*	18 (36.7%)		
*Pembrolizumab*	16 (32.7%)		
*Ipilimumab*	3 (6.1%)		
*Ipilimumab and nivolumab*	11 (22.4%)		
Ipilimumab or nivolumab*	1 (2.0%)		
Second ICI therapy, n (%)			
*Nivolumab*	8 (66.7%)		
*Pembrolizumab*	1 (8.3%)		
*Ipilimumab*	1 (8.3%)		
*Ipilimumab and nivolumab*	2 (16.7%)		
Amount of cycles (total**), median [IQR]	14 [8.5–24.5]		
Time since last cycle (years), median [IQR]	3.7 [2.9–5.1]		

^BMI: body mass index, ICI: immune checkpoint inhibitor, IQR: interquartile range, n: number, NSCLC: non−small cell lung cancer, RCC: renal cell carcinoma, UCC: Urothelial cell carcinoma^

^* Participant in randomized study^

^** Including second ICI^

### Taste and smell tests

**Fig. 1:**
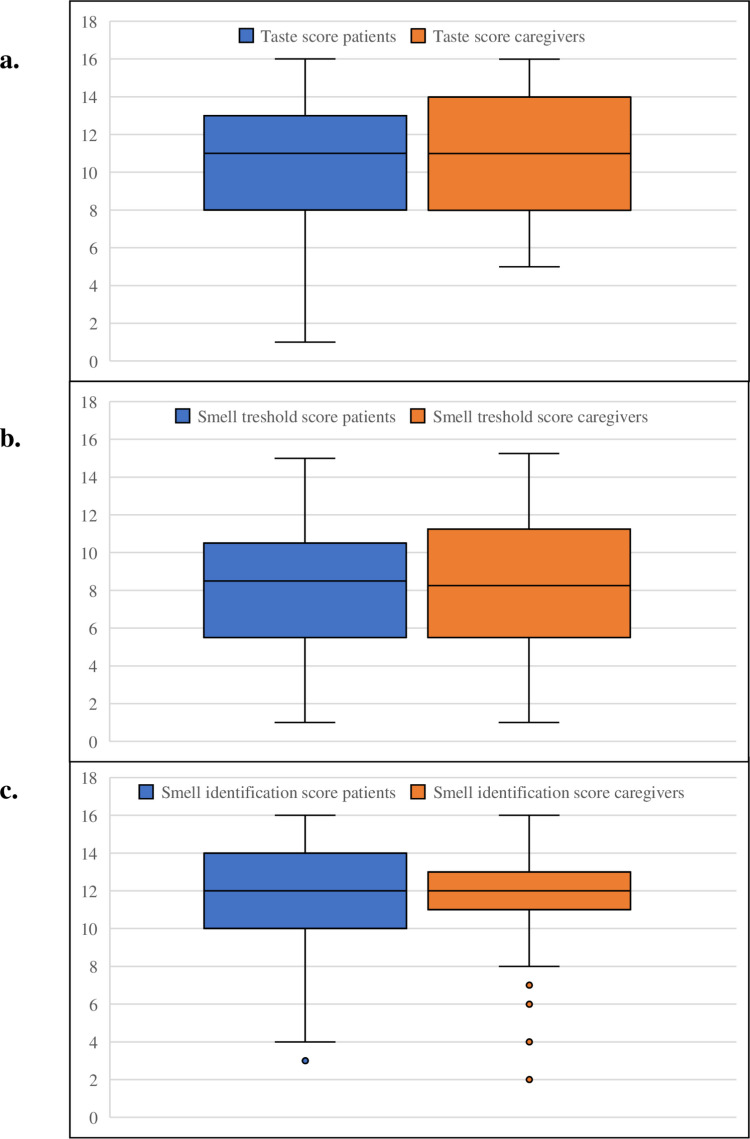
Taste, smell threshold and smell identification scores of patients treated for cancer with immune checkpoint inhibitors and caregivers. Boxplot of A) taste scores, B) smell threshold and C) smell identification scores of patients treated for cancer (blue) and caregivers (red). The boxes represent the interquartile range (IQR), with the median indicated by a horizontal line. The plots illustrate the distribution of scores across groups

For the taste test, no difference was observed in the total taste scores between patients and caregivers (median 11, IQR 8–13 vs. median 11, IQR 8–14; p = 0.37) (Fig. 1). Hypogeusia, indicated by scores below 9, was observed in 31% (*n *= 15) of patients and 29% (*n* = 14)of caregivers.

Hyposmia, defined by an identification score < 11 or a threshold score < 5.8, was observed in 29% (*n* = 14) and 29% (*n* = 14) of the patients and in 20% (*n *= 10) and 27% (*n* = 13) of the caregivers, respectively. Neither significant differences between patients and caregivers were found in the smell identification scores (median 12, IQR 8–13 vs. median 12, IQR 11–13; p = 0.82), nor in the smell threshold scores (median 8.5, IQR 5.3–10.6 vs. median 8.3, IQR 5.4–11.5; p = 0.62) (Fig. 1).

A statistically significant correlation was observed between the smell threshold test and the smell identification test (Spearman’s rho = 0.591, p < 0.001). In addition, when considering all participants together, the taste test correlated weakly with both the smell threshold test (*r *= 0.213, *p* = 0.035) and the smell identification test (*r *= 0.379, p < 0.001).

### Saliva flow rate and composition

**Fig. 2:**
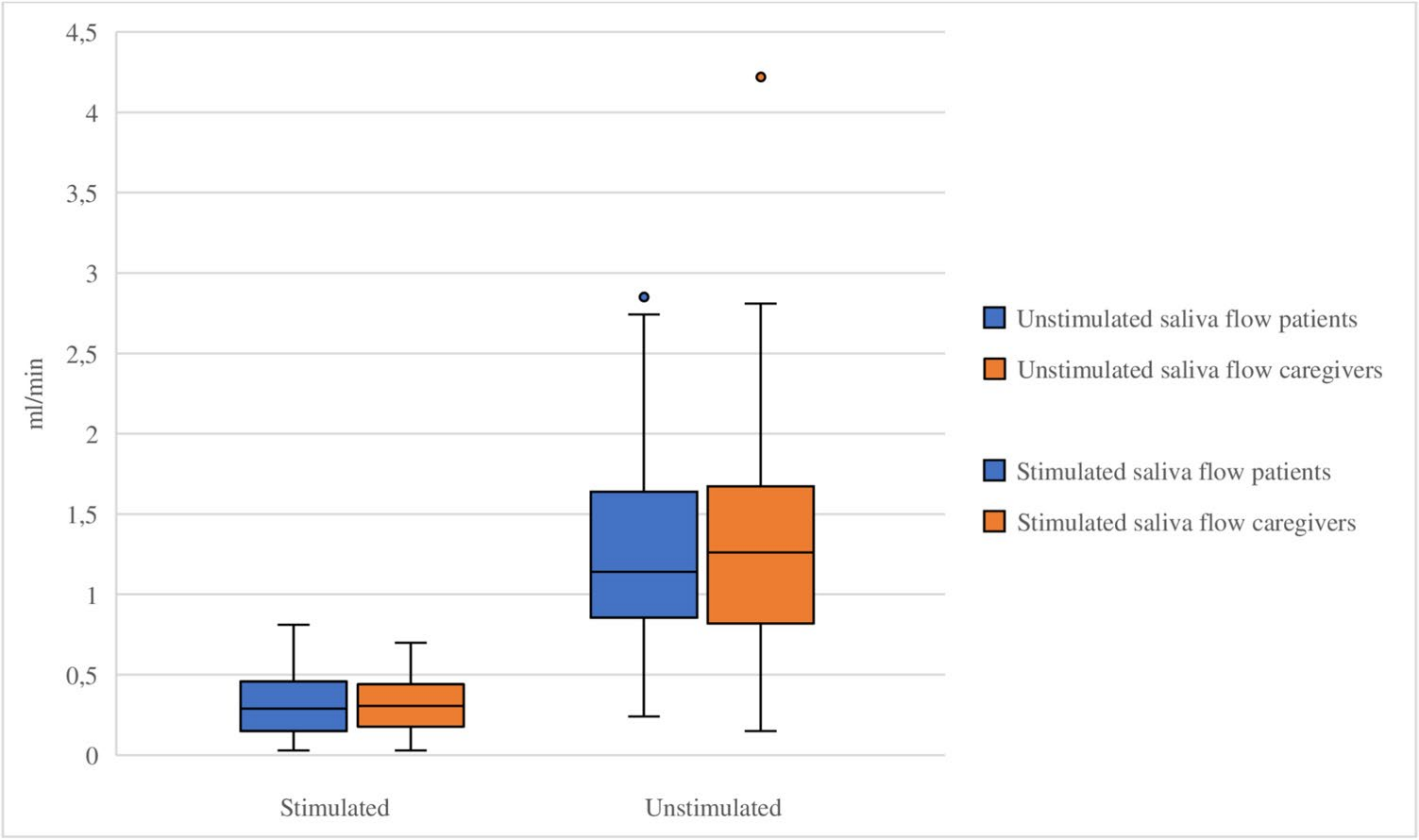
Unstimulated and stimulated saliva flow of patients treated for cancer with immune checkpoint inhibitors and caregivers. Boxplot of unstimulated and stimulated saliva flow in ml/min of patients treated for cancer (blue) and caregivers (red). The boxes represent the interquartile range (IQR), with the median indicated by a horizontal line. The plots illustrate the distribution of scores across groups

Based on the unstimulated saliva flow rate, hyposalivation (< 0.1 mL/min) was observed in 12% (*n* = 6) of the patients, and 6% (*n* = 3) of the caregivers. The difference in unstimulated saliva flow rate between patients and caregivers was not statistically significant (median 0.29 mL/min, IQR 0.15–0.46 vs. median 0.31 mL/min, IQR 0.18–0.45; *p* = 0.97). For stimulated saliva flow rate, hyposalivation (< 0.5 mL/min) was detected in 4% (*n* = 2) of patients, and 6% (*n* = 3) of the caregivers. No significant difference was found between flow rates in patients and caregivers (median 1.14, IQR 0.83–1.65 vs. median 1.26, IQR 0.80–1.69; *p* = 0.87) (Fig. 2). A moderate correlation was found between the unstimulated and stimulated saliva flow rates (*r* = 0.619, p < 0.001) across all participants.

Due to insufficient saliva volume produced by some participants (< 0.5 mL in 5 min), the composition of unstimulated saliva could only be analyzed in 45 patients and 45 caregivers. To account for differences in salivary flow rate, the saliva concentrations were adjusted for flowrate by transforming to absolute amounts excreted (output per minute). After this transformation, only sodium was significantly higher in patients (*p *= 0.027), while differences in potassium (*p *= 0.106), ammonium (*p* = 0.066), magnesium (*p *= 0.065), calcium (*p* = 0.862), and total protein (*p *= 0.076) were not statistically different. Table 2 shows both The concentration-based saliva analysis is shown in Table 2 part A and the flow-rate adjusted secretion rates are given in part B.

**Table 2:** Composition of unstimulated whole saliva of patients treated with immune checkpoint inhibitors and caregivers: (a) concentration-based analysis and (b) flow rate–adjusted secretion rates

Salivary component	Patients n = 45*	Caregivers n = 45*	P-value
A			
Sodium (mg/L), mean ± SD	14.99 ± 6.96	11.30 ± 5.09	0.003
Potassium (mg/L), mean ± SD	80.08 ± 25.07	70.97 ± 17.33	0.017
Ammonium (mg/L), mean ± SD	11.77 ± 5.89	9.34 ± 5.07	0.019
Magnesium (mg/L), median [IQR]	0.538 [0.428–0.628]	0.416 [0.315–0.569]	0.007
Calcium (mg/L), median [IQR]	2.25 [1.96–3.58]	2.56 [1.97–3.36]	0.672
Total protein (µg/ml), mean ± SD	1803 ± 607	1565 ± 563	0.028
Amylase (U/ml), mean ± SD	376.0 ± 203.3	413.1 ± 226.0	0.208
Mucin5B (mg/ml), median [IQR]	0.816 [0.472–1.476]	0.564 [0.284–1.244]	0.069
B			
Sodium (mg/L), mean ± SD	4.837 ± 3.451	3.665 ± 2.066	0.027
Potassium (mg/L), mean ± SD	26.97 ± 17.31	23.05 ± 11.86	0.106
Ammonium (mg/L), mean ± SD	3.930 ± 3.267	3.037 ± 2.210	0.066
Magnesium (mg/L), median [IQR]	0.168 [0.107–0.251]	0.127 [0.096–0.167]	0.065
Calcium (mg/L), median [IQR]	0.851 [0.511–1.149]	0.759 [0.535–1.081]	0.862
Total protein (µg/ml), mean ± SD	606.1 ± 419.1	498.6 ± 271.0	0.076
Amylase (U/ml), mean ± SD	133.1 ± 134.1	128.9 ± 81.0	0.429
Mucin5B (mg/ml), median [IQR]	0.209 [0.122–0.450]	0.162 [0.061–0.353]	0.364

IQR: interquartile range, SD: standard deviation. * Only participants with adequate saliva volume (>0.5 mL) could be analyzed.

### Questionnaires

No difference in prevalence of risk of malnutrition was observed between patients and caregivers (*p* = 0.092). The median was 0.0 [0.0–1.0] in both groups. Moreover, none of the participants had a score of nine or higher, indicating that none of them were at a high risk of malnutrition.

**Fig. 3:**
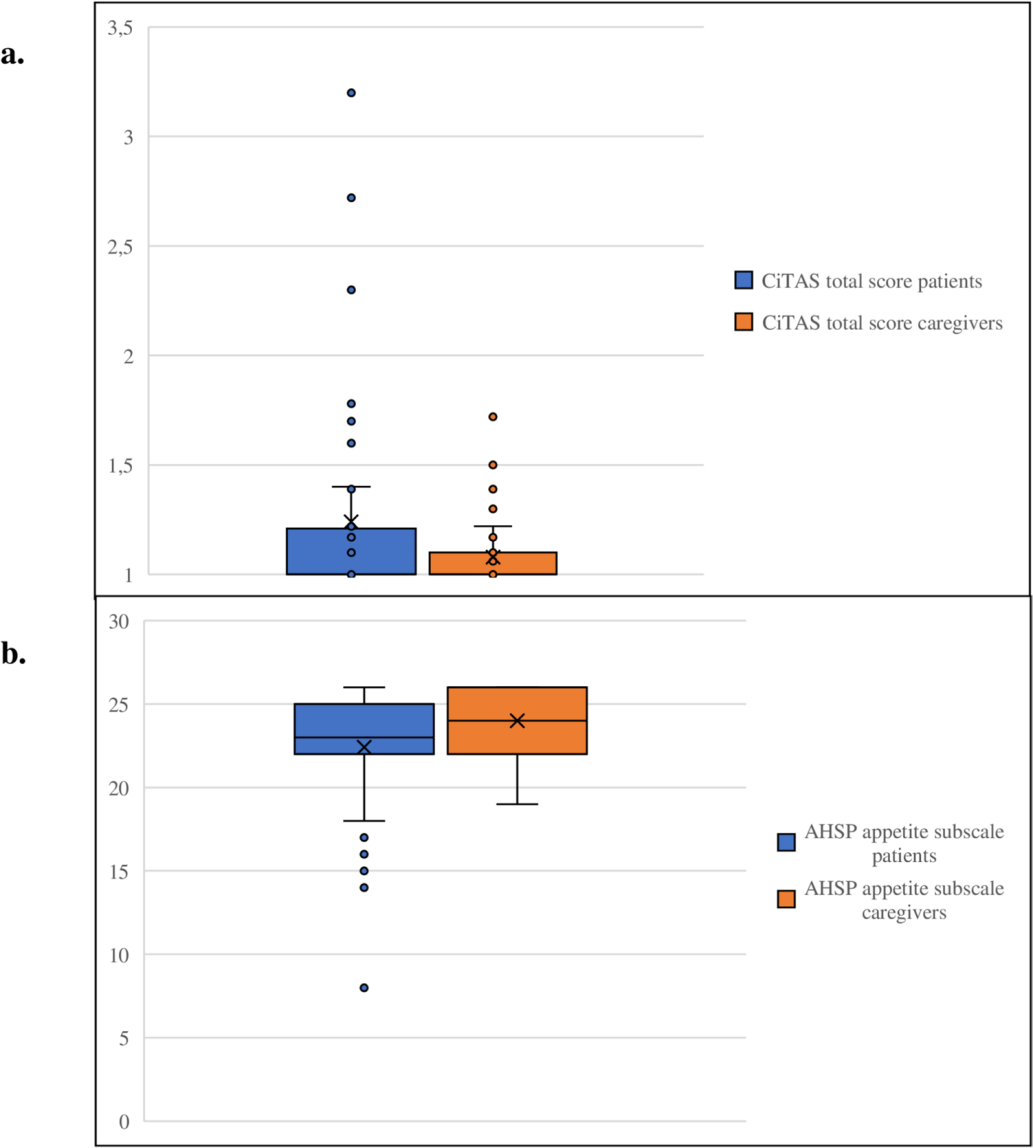
CiTAS total score and AHSP appetite subscale score of patients treated with immune checkpoint inhibitors and caregivers. Boxplot of A) CiTAS total score and B) AHSP appetite subscale score of patients treated for cancer (blue) and caregivers (red). The boxes represent the interquartile range (IQR), with the median indicated by a horizontal line. The plots illustrate the distribution of scores across groups

Regarding subjective taste dysfunction, patients scored higher than caregivers on the CiTAS total score (p = 0.040) (Fig. 3A). Additional differences were found for the CiTAS subscales ‘basic tastes’ (*p* = 0.024), ‘discomfort’ (*p* = 0.028), and ‘phantogeusia or parageusia’ (*p* = 0.048). In contrast, no significant difference was observed for the subscale ‘general taste changes’ (*p* = 0.174). Six patients (12%) reported mild taste alterations and two (4%) moderate alterations, whereas only two caregivers (4%) reported mild alterations. The total CiTAS score did not correlate significantly with the objective taste test (*r* = –0.107, *p* = 0.30), although two patients with moderate scores also had objective hypogeusia.

For appetite and sensory perception, patients scored significantly lower than caregivers on the AHSP appetite subscale (*p* = 0.017) (Fig. 3B). No significant differences were observed for the AHSP subscales taste (*p* = 0.165), smell (*p* = 0.997), or craving (*p *= 0.059).

No significant difference in the severity of xerostomia, as measured by the XI questionnaire, was found between patients and caregivers (*p* = 0.13). However, for the RODI questionnaire, significant differences were found for two of nine intra-oral areas: anterior palate (*p* = 0.037) and anterior tongue (*p* = 0.040). A weak correlation was observed between unstimulated saliva flow rates and XI questionnaire scores (*r* = −0.223,* p* = 0.029). No significant correlation was found between stimulated saliva flow rate and XI scores (*r* = −0.108, *p* = 0.30).

A subgroup of 22 patients and 20 caregivers indicated experiencing taste and/or smell changes. In this subgroup, a difference in QoL scores was found for two specific questions in the QOD questionnaire: Q12 (‘I experience more stress than before due to my olfactory and/or gustatory disorder’) (*p* = 0.016), and Q13 (‘Due to my olfactory and/or gustatory disorder, I sometimes have thoughts or ideas that I prefer not to share with others’) (*p* = 0.033).

Online Resource 1 presents the median scores for patients and caregivers for the domains of all questionnaires (without QOD).

### Correlations between taste and smell tests, saliva and questionnaires

No significant correlations were found between the time since the last ICI treatment, type of ICI or the total number of ICI cycles, objective taste and smell changes, and stimulated and unstimulated saliva flow rates.

## Discussion

To gain more insight in the prevalence and characteristics of taste and smell alterations, and xerostomia after ICI treatment, this study examined a cohort of patients who had received ICI therapy for different tumor types more than two years ago, and caregivers. Objective taste and smell function, stimulated and unstimulated saliva flow rate, and xerostomia did not differ between patients treated for cancer and caregivers. Some differences were found in subjective taste perception. Furthermore, patients scored lower on the appetite subscale of the AHSP questionnaire, and higher on sodium concentration compared to caregivers.

This study confirms the presence of objective taste and smell dysfunction, in respectively 31% and 29% of the patients, but does not show a significant difference between patients and caregivers. Previous studies have reported varying prevalences of dysgeusia during treatment with ICIs, i.e., 3 to 24% [13–16]. However, no studies have reported on dysgeusia after treatment with ICIs. The finding that a subgroup of participants in both groups reported taste and smell dysfunction suggests that these symptoms occur independently of ICI treatment. ICI treatment does not appear to increase this risk, which is an important and reassuring finding, especially in contrast to treatment with chemotherapy and radiotherapy, which are known to cause permanent taste and smell alterations [42].

Although several (sub)scales of the CiTAS and the appetite subscale of the AHSP questionnaire showed differences between patients and caregivers, the median scores across groups were nearly identical. This suggests that these differences may not be clinically relevant and are likely caused by a small number of outliers. The finding that patients reported more subjective taste disturbances than caregivers, without differences in objective taste function, is consistent with previous studies [42]. One possible explanation is that objective taste tests focus on basic taste detection at established thresholds, but may overlook complex alterations like phantom tastes or dysgeusia, which are frequently reported by patients treated for cancer and captured by the CiTAS questionnaire [24, 43].

The comparable prevalence of xerostomia between patients treated for cancer and caregivers is inconsistent with prior studies [44]. For instance, a retrospective cohort study in patients with stage III to IV melanomas treated with anti-PD-1 showed that 53% of patients developed xerostomia that persisted beyond 12 weeks after ICI discontinuation [12]. In contrast, a recent meta-analysis estimated the pooled prevalence of xerostomia to be only 5% in clinical trials [16]. However, the results of the present study are difficult to compare directly with these findings, as different methods have been used to determine the subjective sensation of a dry mouth. In the present study, an internationally validated questionnaire was used to quantify the severity of xerostomia [37]. In addition, the present study focused on long-term survivors more than two years after ICI treatment. This suggests that xerostomia may resolve over time in some patients, although long-term prevalence estimates vary depending on the methods used to assess it. While clinical trials often underreport mild or subjective symptoms, observational studies may provide a more accurate picture of the prevalence and persistence of xerostomia in practice.

In the analysis of saliva composition, after correction for salivary flow rate, only sodium output remained significantly higher in the patients compared to the caregivers. Elevated salivary sodium is considered a marker of impaired gland function, reflecting reduced water secretion and potentially contributing to xerostomia, caries, and oral discomfort [45]. For ammonium, magnesium, and total protein, differences that were initially significant disappeared after correction for salivary flow rate, suggesting that these changes were largely driven by dilution effects rather than (minor) impaired gland function. However, the persistent difference in sodium output after correction suggests that factors beyond dilution may be involved.

The findings of this study suggest that chronic immune-related adverse events are not uniformly present among long-term responders to immunotherapy. The mechanisms underlying chronic immune-related adverse events remain poorly understood. It has been suggested that irreversible tissue damage or persistent low-grade inflammation may result from toxicity [46]. Interestingly, previous studies have shown a correlation between the occurrence of immune-related AEs and improved survival outcomes, suggesting a possible link between immune activation and therapeutic benefit [46, 47]. This present study included long-term survivors who are no longer receiving treatment, have responded well to immunotherapy, and lack substantial oral toxicity. This suggests that not all long-term responders experience such effects, underscoring the heterogeneity of chronic immune-related AEs and highlighting the need for further research into patient-specific risk factors.

One of the main strengths of this study is its broad approach to assess taste and smell function in patients treated with ICIs and control subjects by including both objective sensory tests and subjective questionnaires. Furthermore, the inclusion of a control group of caregivers provided insight into whether observed sensory changes are specific to patients treated with ICIs or comparable to the general population. These caregivers were mainly partners and relatives of the patients treated for cancer, and therefore may share certain lifestyle factors. However, future research should also use a prospective design to follow participants over time to better understand how taste and smell functions evolve and disappear in individual patients. Another strength of this study is the patient population. The inclusion of a heterogeneous group with different types of tumors and different ICIs provides a broad overview of taste and smell dysfunction, as well as xerostomia, more than two years after ICI therapy. Additionally, a strength is that only patients who exclusively underwent ICI therapy, without any other systemic treatments, were included. This provided a clear picture of the effects of ICIs alone.

The current study also has some limitations. Firstly, the study was performed after the COVID-19 pandemic and COVID-19-related sensory alterations could be present in the participants. Although we asked participants whether they had experienced COVID-19, not everyone may have been aware of an infection and the taste and smell dysfunction it may have caused. Secondly, a limitation is the possible selection bias in the study. Participants were screened to exclude those with any prior taste or smell disorders, which may have led to a distorted representation of the population, as taste and smell disorders occur in 17% and 14%, respectively, of the general population[48]. Thirdly, it is important to note that salivary composition was only analyzed in participants with sufficient saliva volume. Those with very low unstimulated flow rates could not be included due to sample limitations, which may have influenced the findings by excluding individuals with potentially more pronounced glandular dysfunction. Therefore, the results may underestimate the full extent of changes in composition in patients with the lowest saliva production.

In the future, qualitative studies exploring the personal and psychosocial impact of these sensory alterations may serve as a guide for supportive care strategies. It is also important to investigate the effects of interventions, such as dietary modifications, saliva substitutes, or taste and smell training, to reduce symptoms and improve quality of life.

To conclude, this study provides largely reassuring findings for patients who will start ICI therapy. No long-term differences were observed in objective taste and smell function, salivary flow rates, or severity of xerostomia in patients at least two years after ICI therapy compared to caregivers. Although some patients reported subjective sensory disturbances and minor differences in saliva composition were detected, these findings appear to be limited to a small subgroup and may not be clinically relevant. Moreover, it remains unclear to what extent these changes can be attributed to prior ICI treatment, given the absence of consistent objective abnormalities. Nonetheless, clinicians should pay attention to sensory complaints, as these can impact QoL, even when objective tests show no abnormalities.

## Supplementary Information

Below is the link to the electronic supplementary material.

ESM 1 (DOCX 17.1 KB)

## Data Availability

The data is available on request.

## References

[1] Bagchi S, Yuan R, Engleman EG (2021) Immune checkpoint inhibitors for the treatment of cancer: clinical impact and mechanisms of response and resistance. Annu Rev Pathol Mech Dis 16:223–249. 10.1146/annurev-pathol-042020-042741 10.1146/annurev-pathol-042020-042741 33197221

[2] van den Bulk J, Verdegaal EM, de Miranda NF (2018) Cancer immunotherapy: broadening the scope of targetable tumours. Open Biol 8:180037. 10.1098/rsob.180037 29875199 10.1098/rsob.180037 PMC6030119

[3] Darvin P, Toor SM, Sasidharan Nair V, Elkord E (2018) Immune checkpoint inhibitors: recent progress and potential biomarkers. Exp Mol Med 50:1–11. 10.1038/s12276-018-0191-1 30546008 10.1038/s12276-018-0191-1 PMC6292890

[4] Wolchok JD, Vanna C-S, Piotr R et al (2025) Final, 10-year outcomes with Nivolumab plus Ipilimumab in advanced melanoma. N Engl J Med 392:11–22. 10.1056/NEJMoa2407417 39282897 10.1056/NEJMoa2407417 PMC12080919

[5] Chapman PB, Einhorn LH, Meyers ML et al (2026) Phase III multicenter randomized trial of the Dartmouth regimen versus Dacarbazine in patients with metastatic melanoma. J Clin Oncol 17:2745. 10.1200/JCO.1999.17.9.2745 10.1200/JCO.1999.17.9.2745 10561349

[6] Johnson DB, Nebhan CA, Moslehi JJ, Balko JM (2022) Immune-checkpoint inhibitors: long-term implications of toxicity. Nat Rev Clin Oncol 19:254–267. 10.1038/s41571-022-00600-w 35082367 10.1038/s41571-022-00600-w PMC8790946

[7] Jin Y, Wei J, Weng Y et al (2022) Adjuvant therapy with PD1/PDL1 inhibitors for human cancers: a systematic review and meta-analysis. Front Oncol 12:732814. 10.3389/fonc.2022.732814 35280727 10.3389/fonc.2022.732814 PMC8913885

[8] Topalian SL, Forde PM, Emens LA et al (2023) Neoadjuvant immune checkpoint blockade: a window of opportunity to advance cancer immunotherapy. Cancer Cell 41:1551–1566. 10.1016/j.ccell.2023.07.011 37595586 10.1016/j.ccell.2023.07.011 PMC10548441

[9] Chhabra N, Kennedy J (2021) A review of cancer immunotherapy toxicity: immune checkpoint inhibitors. J Med Toxicol 17:411–424. 10.1007/s13181-021-00833-8 33826117 10.1007/s13181-021-00833-8 PMC8455777

[10] Postow MA, Sidlow R, Hellmann MD (2018) Immune-related adverse events associated with immune checkpoint blockade. N Engl J Med 378:158–168. 10.1056/NEJMra1703481 29320654 10.1056/NEJMra1703481

[11] Topalian SL, Hodi FS, Brahmer JR et al (2019) Five-year survival and correlates among patients with advanced melanoma, renal cell carcinoma, or non-small cell lung cancer treated with Nivolumab. JAMA Oncol 5:1411–1420. 10.1001/jamaoncol.2019.2187 31343665 10.1001/jamaoncol.2019.2187 PMC6659167

[12] Patrinely JR Jr, Johnson R, Lawless AR et al (2021) Chronic immune-related adverse events following adjuvant anti–PD-1 therapy for high-risk resected melanoma. JAMA Oncol 7:744–748. 10.1001/jamaoncol.2021.0051 33764387 10.1001/jamaoncol.2021.0051 PMC7995124

[13] Vigarios E, Epstein JB, Sibaud V (2017) Oral mucosal changes induced by anticancer targeted therapies and immune checkpoint inhibitors. Support Care Cancer 25:1713–1739. 10.1007/s00520-017-3629-4 28224235 10.1007/s00520-017-3629-4

[14] Srivastava A, Al-Zubidi N, Appelbaum E et al (2020) Immune-related oral, otologic, and ocular adverse events. Adv Exp Med Biol 1244:295–307. 10.1007/978-3-030-41008-7_17 32301024 10.1007/978-3-030-41008-7_17

[15] Xu Y, Wen N, Sonis ST, Villa A (2021) Oral side effects of immune checkpoint inhibitor therapy (ICIT): an analysis of 4683 patients receiving ICIT for malignancies at Massachusetts General Hospital, Brigham & Women’s Hospital, and the Dana-Farber Cancer Institute, 2011 to 2019. Cancer 127:1796–1804. 10.1002/cncr.33436 33595843 10.1002/cncr.33436

[16] Srivastava A, Nogueras-Gonzalez GM, Geng Y et al (2024) Oral toxicities associated with immune checkpoint inhibitors: meta-analyses of clinical trials. J Immunother Precis Oncol 7:24–40. 10.36401/JIPO-23-14 38327757 10.36401/JIPO-23-14 PMC10846637

[17] Huisman F, Van Elst JM, Reyners AKL et al (2021) Taste and smell changes resulting from cancer therapies. Ned Tijdschr Geneeskd 165:D5696 35129889

[18] Zabernigg A, Gamper E-M, Giesinger JM et al (2010) Taste alterations in cancer patients receiving chemotherapy: a neglected side effect? Oncologist 15:913–920. 10.1634/theoncologist.2009-0333 20667968 10.1634/theoncologist.2009-0333 PMC3228016

[19] Bustillos H, Indorf A, Alwan L et al (2022) Xerostomia: an immunotherapy-related adverse effect in cancer patients. Support Care Cancer 30:1681–1687. 10.1007/s00520-021-06535-9 34562169 10.1007/s00520-021-06535-9

[20] Rehak NN, Cecco SA, Csako G (2000) Biochemical composition and electrolyte balance of “unstimulated” whole human saliva. Clin Chem Lab Med 38:335–343. 10.1515/CCLM.2000.049 10928655 10.1515/CCLM.2000.049

[21] Jensen SB, Pedersen AML, Vissink A et al (2010) A systematic review of salivary gland hypofunction and xerostomia induced by cancer therapies: prevalence, severity and impact on quality of life. Support Care Cancer 18:1039–1060. 10.1007/s00520-010-0827-8 20237805 10.1007/s00520-010-0827-8

[22] Lan X, Chan JYK, Pu JJ et al (2020) Saliva electrolyte analysis and xerostomia-related quality of life in nasopharyngeal carcinoma patients following intensity-modulated radiation therapy. Radiother Oncol 150:97–103. 10.1016/j.radonc.2020.06.016 32544605 10.1016/j.radonc.2020.06.016

[23] Brisbois TD, de Kock IH, Watanabe SM et al (2011) Characterization of chemosensory alterations in advanced cancer reveals specific chemosensory phenotypes impacting dietary intake and quality of life. J Pain Symptom Manage 41:673–683. 10.1016/j.jpainsymman.2010.06.022 21276701 10.1016/j.jpainsymman.2010.06.022

[24] Postma EM, de Vries YC, Boesveldt S (2017) [Tasty food for cancer patients: the impact of smell and taste alterations on eating behaviour]. Ned Tijdschr Geneeskd 160:D748 28074724

[25] de Vries YC, Boesveldt S, Kelfkens CS et al (2018) Taste and smell perception and quality of life during and after systemic therapy for breast cancer. Breast Cancer Res Treat 170:27–34. 10.1007/s10549-018-4720-3 29476290 10.1007/s10549-018-4720-3 PMC5993854

[26] Navazesh M, Kumar SKS (2008) Measuring salivary flow: challenges and opportunities. J Am Dent Assoc 139:35S-40S. 10.14219/jada.archive.2008.0353 18460678 10.14219/jada.archive.2008.0353

[27] Villa A, Connell C, Abati S (2014) Diagnosis and management of xerostomia and hyposalivation. Ther Clin Risk Manag 11:45–51. 10.2147/TCRM.S76282 25653532 10.2147/TCRM.S76282 PMC4278738

[28] Landis BN, Welge-Luessen A, Brämerson A et al (2009) “Taste Strips” - a rapid, lateralized, gustatory bedside identification test based on impregnated filter papers. J Neurol 256:242–248. 10.1007/s00415-009-0088-y 19221845 10.1007/s00415-009-0088-y

[29] Hummel T, Sekinger B, Wolf SR et al (1997) “Sniffin” sticks’: olfactory performance assessed by the combined testing of odor identification, odor discrimination and olfactory threshold. Chem Senses 22:39–52. 10.1093/chemse/22.1.39 9056084 10.1093/chemse/22.1.39

[30] burghart messtechnik (2025) Odofin Sniffin’ Sticks Identification Test. https://www.burghart-mt.de/daten/www.burghart-mt.de/datei/de/ident_test_207.pdf. Accessed 21 April 2026.

[31] burghart messtechnik (2025) Odofin Sniffin’ Sticks Threshold Test. https://www.burghart-mt.de/daten/www.burghart-mt.de/datei/de/schwellentest_318.pdf. Accessed 21 April 2026.

[32] Noort H, Cruijsen E, Pie R, et al (2020) CiTAS-NL: Smaakveranderingen bij chemotherapie gemeten. Ned Tijdschr Voeding Diëtetiek 5.

[33] Jager-Wittenaar H, Ottery FD (2017) Assessing nutritional status in cancer: role of the Patient-Generated Subjective Global Assessment. Curr Opin Clin Nutr Metab Care 20:322–329. 10.1097/MCO.0000000000000389 28562490 10.1097/MCO.0000000000000389

[34] Sealy MJ, Haß U, Ottery FD et al (2018) Translation and cultural adaptation of the Scored Patient-Generated Subjective Global Assessment: an interdisciplinary nutritional instrument appropriate for Dutch cancer patients. Cancer Nurs 41:450–462. 10.1097/NCC.0000000000000505 28538001 10.1097/NCC.0000000000000505

[35] Mathey MF (2001) Assessing appetite in Dutch elderly with the appetite, hunger and sensory perception (AHSP) questionnaire. J Nutr Health Aging 5:22–28 11250665

[36] Frasnelli J, Hummel T (2005) Olfactory dysfunction and daily life. Eur Arch Otorhinolaryngol 262:231–235. 10.1007/s00405-004-0796-y 15133691 10.1007/s00405-004-0796-y

[37] Thomson WM, Chalmers JM, Spencer AJ, Williams SM (1999) The xerostomia inventory: a multi-item approach to measuring dry mouth. Community Dent Health 16:12–17 10697349

[38] Assy Z, Bots CP, Arisoy HZ et al (2021) Differences in perceived intra-oral dryness in various dry-mouth patients as determined using the regional oral dryness inventory. Clin Oral Investig 25:4031–4043. 10.1007/s00784-020-03734-2 33496869 10.1007/s00784-020-03734-2 PMC8137633

[39] Faruque M, Nazmi K, van Splunter A et al (2023) Sialagogic effects through olfactory stimulation with mastic resin and α-pinene volatiles in vivo. Biomed Pharmacother 168:115699. 10.1016/j.biopha.2023.115699 37865987 10.1016/j.biopha.2023.115699

[40] Potocka W, Assy Z, van Splunter AP et al (2025) From scent to saliva: the saliva-stimulating effect of terpenes in dry mouth. Arch Oral Biol 175:106282. 10.1016/j.archoralbio.2025.106282 40347848 10.1016/j.archoralbio.2025.106282

[41] Al Habobe H, Haverkort EB, Nazmi K et al (2024) The impact of saliva collection methods on measured salivary biomarker levels. Clin Chim Acta 552:117628. 10.1016/j.cca.2023.117628 37931731 10.1016/j.cca.2023.117628

[42] Spotten LE, Corish CA, Lorton CM et al (2017) Subjective and objective taste and smell changes in cancer. Ann Oncol 28:969–984. 10.1093/annonc/mdx018 28327968 10.1093/annonc/mdx018

[43] IJpma I, Timmermans ER, Renken RJ et al (2017) Metallic taste in cancer patients treated with systemic therapy: a questionnaire-based study. Nutr Cancer 69:140–145. 10.1080/01635581.2017.1250922 27925850 10.1080/01635581.2017.1250922

[44] Warner BM, Baer AN, Lipson EJ et al (2019) Sicca syndrome associated with immune checkpoint inhibitor therapy. Oncologist 24:1259–1269. 10.1634/theoncologist.2018-0823 30996010 10.1634/theoncologist.2018-0823 PMC6738284

[45] Pedersen AML, Bardow A, Nauntofte B (2005) Salivary changes and dental caries as potential oral markers of autoimmune salivary gland dysfunction in primary Sjögren’s syndrome. BMC Clin Pathol 5:4. 10.1186/1472-6890-5-4 15740617 10.1186/1472-6890-5-4 PMC554998

[46] Fletcher K, Johnson DB (2024) Chronic immune-related adverse events arising from immune checkpoint inhibitors: an update. J Immunother Cancer 12:e008591. 10.1136/jitc-2023-008591 38964785 10.1136/jitc-2023-008591 PMC11227828

[47] Das S, Johnson DB (2019) Immune-related adverse events and anti-tumor efficacy of immune checkpoint inhibitors. J Immunother Cancer 7:306. 10.1186/s40425-019-0805-8 31730012 10.1186/s40425-019-0805-8 PMC6858629

[48] Liu G, Zong G, Doty RL, Sun Q (2016) Prevalence and risk factors of taste and smell impairment in a nationwide representative sample of the US population: a cross-sectional study. BMJ Open 6:e013246. 10.1136/bmjopen-2016-013246 28157672 10.1136/bmjopen-2016-013246 PMC5129069

