# Supplementary material for: Prevalence and characteristics of persistent taste and smell dysfunction after immune checkpoint inhibitor therapy for cancer
Source: Support Care Cancer. 2026 May 21;34(6):564. doi: 10.1007/s00520-026-10761-4 (PMC13194279; doi:10.1007/s00520-026-10761-4)
Supplement: Supplementary file 1 — (DOCX 17.1 KB) [file 520_2026_10761_MOESM1_ESM.docx]

**Supplementary files**

**Title:** Persistent taste and smell dysfunction after immune checkpoint inhibitor therapy for cancer

**Joural name:** Supportive Care in Cancer

**Authors:**

Jip M. van Elst, Corine Buffinga, Henk S. Brand, Lucie B.M. Hijmering-Kappelle, Harriët Jager-Wittenaar, Anna K.L. Reyners, Janine Nuver, Jacco J. de Haan*

*Corresponding author: J.J. de Haan, MD, PhD. E-mail: j.j.de.haan@umcg.nl

| **Questionnaire** | **Patients n=49** | **Caregivers**  **n=49** | **P-value** |
| --- | --- | --- | --- |
| PG-SGA SF score, median [IQR] | 0.0 [0.0-1.0] | 0.0 [0.0-1.0] | 0.092 |
| CiTAS total score, median [IQR]  CiTAS basic tastes  CiTAS discomfort  CiTAS phantogeusia or parageusia  CiTAS general taste changes | 1.0 [1.0-1.2]  1.0 [1.0-1.1]  1.0 [1.0-1.3]  1.0 [1.0-1.0]  1.0 [1.0-1.5] | 1.0 [1.0-1.1]  1.0 [1.0-1.0]  1.0 [1.0-1.0]  1.0 [1.0-1.0]  1.0 [1.0-1.5] | 0.040  0.024  0.028  0.048  0.174 |
| AHSP appetite, median [IQR]  AHSP taste  AHSP smell  AHSP craving | 23.0 [22.0-25.0]  29.0 [27.0-31.0]  22.0 [19.0-24.0]  36.0 [34.0-38.8] | 24 [22.0-26.0]  30 [28.0-31.5]  22 [18.5-24.0]  38.0 [35.0-39.5] | 0.017  0.165  0.997  0.059 |
| XI total score, median [IQR] | 18.0 [15.0-25.0] | 17.0 [14.0-20.0] | 0.132 |
| RODI upper lip, median [IQR]  RODI front part of palate  RODI inside of the cheeks  RODI back part of the palate  RODI lower lip  RODI floor of the mouth  RODI back part of the tongue  RODI front part of the tongue  RODI throat | 2.0 [1.0-2.0]  1.0 [1.0-2.0]  1.0 [1.0-1.0]  1.0 [1.0-2.0]  2.0 [1.0-2.0]  1.0 [1.0-2.0]  1.0 [1.0-2.0]  1.0 [1.0-2.0]  1.5 [1.0-2.0] | 2.0 [1.0-2.0]  1.0 [1.0-1.0]  1.0 [1.0-1.0]  1.0 [1.0-1.0]  1.0 [1.0-2.0]  1.0 [1.0-1.0]  1.0 [1.0-1.0]  1.0 [1.0-1.0]  1.0 [1.0-2.0] | 0.924  0.037  0.354  0.440  0.426  0.087  0.312  0.040  0.152 |

**Table S1** – Subjective taste and smell changes, and xerostomia of patients treated with immune checkpoint inhibitors and caregivers

AHSP: Appetite, Hunger, and Sensory Perception, CiTAS: Chemotherapy-induced Taste Alteration Scale, IQR: interquartile range, n: number, PG-SGA SF: Patient-Generated Subjective Global Assessment Short Form, QOD: Questionnaire of Olfactory Disorders, RODI: Regional Oral Dryness Inventory, XI: Xerostomia Inventory.
